# Supplementary material for: The impact of the COVID-19 pandemic on the mental health of families dealing with attention-deficit hyperactivity disorder
Source: PLoS One. 2023 Mar 16;18(3):e0283227. doi: 10.1371/journal.pone.0283227 (PMC10019744; doi:10.1371/journal.pone.0283227)
Supplement: S1 Appendix — (DOCX) [file pone.0283227.s001.docx]

**S1 Appendix – Parent Interview Guide**

**Interviewer:** *Today, I am going to ask you how the COVID-19 pandemic has impacted your child and family*.

**Part 1** – **Interviewer**: *When thinking of your child(ren) with ADHD*…

1. Have there been unique barriers for your child during the COVID-19 pandemic because of their ADHD diagnosis?
2. Have you noticed any changes in the presenting characteristics or symptoms associated with your child’s ADHD?

**Part 2 – Interviewer**: *The next few questions aim to understand how the COVID-19 pandemic has impacted your child’s mental health and your own mental health*.

1. How was your child’s mental health (overall well-being, temperament, mood) prior to the COVID-19 pandemic?
2. How was your mental health (overall well-being, temperament, mood) prior to the COVID-19 pandemic?
3. Since the COVID-19 pandemic, have you noticed changes in your child’s **temperament**?
   1. **Mood**?
   2. **Anxiety/depression**?
   3. **Energy**?
   4. **Social interactions**?
4. In your opinion, what are the biggest barriers to maintaining optimal mental health for yourself and for your child during the COVID-19 pandemic?
