# Supplementary material for: The impact of the COVID-19 pandemic on the mental health of families dealing with attention-deficit hyperactivity disorder
Source: PLoS One. 2023 Mar 16;18(3):e0283227. doi: 10.1371/journal.pone.0283227 (PMC10019744; doi:10.1371/journal.pone.0283227)
Supplement: Attachment — Submitted filename: Response to Reviewer.docx [file pone.0283227.s003.docx]

**Reviewer #1**

**Comment 1** Thank you very much for adding a theoretical framework to make this paper more academically sound.

**Response:** Thank you for the thoughtful suggestion, we agree that it has made the paper more academically sound.

**Comment 2:** This paper is about ADHD patients in the COVID-19 pandemic. I do believe that an important literature review section is missing in the introduction. Now the introduction includes a) general impact from the COVID-19 pandemic; b) systems theory; c) a very short research gap discussion paragraph. I think the introduction could be made longer. The authors can write a section especially on reviewing literature regarding how ADHD families react to the COVID-19 pandemic or how they are affected by the COVID-19 pandemic. I tried searching “ADHD AND COVID” on Google Scholar, it gave me a lot of interesting relevant results. By reviewing the current findings regarding ADHD and COVID, I am sure that the authors can deepen the introduction section and make their research motivation more strongly justified. To me the research motivation is suggested suddenly in this version of manuscript.

**Response**: We have included a section capturing the recent work that has been done on the impact of COVID-19 among ADHD individuals and families. This begins on line 100.

**Comment 3:** Maybe the section “research gap and objectives” should be put before the section “theory and hypothesis”? is it more sensible?

**Response**: Thank you for this suggestion. We have moved “research gap and objectives” before the “study rationale and theoretical framework” section. We agree that this flow is more sensible.

**Comment 4** The results are not surprising. The authors discuss the findings one by one in the paper. Any synthesis can be made so that more insights that are significant to the field can be suggested?

**Response**: The reporting structure in content analysis is not based on the novelty of findings relative to the literature but based on the frequency of certain themes arising in interviews and on inter-data uniqueness of the themes. While there are commonalities among some themes and subthemes, they each capture unique information that would not be conducive to grouping. Also, each theme/subtheme has an associated and representative quote from participants to offer explicit and nuanced insight into the topics that emerged in the data. We appreciate how the results may seem overly detailed to some, but we strongly advocate for as much detail and transparency in the reporting as possible, even if some of the findings are not novel.

**Comment 5**:  Any theoretical contribution that can be brought by the findings of this paper? For example, how can the findings help us understand the Systems Theory?

**Response**: We have incorporated discussion of the theoretical contributions on page 31 (line 683).

**Comment 6:** To me the Systems Theory is added to the introduction deliberately, it just shows up a bit in the introduction, but it has all been forgotten in the discussion. I think it would be better if the authors can relate back to the theoretical framework in the discussion so that theoretical significance of this paper can be discussed.

**Response**: Thank you for pointing this out. We have incorporated discussion on how the findings relate back to Systems Theory on page 31 (line 683).

**Comment 7**: While the writing is very good, systematic, logical, and easy to follow in this paper, it also provides us with a lot of valuable information about the ADHD families, however the paper is too “practical” and the theoretical discussion is kind of not strong enough.

**Response**: We have incorporated more theoretical discussion starting on pages 31-32. While we agree that discussing more theory is beneficial, and we have gone ahead and done so, a major goal of this work was to offer insight into how families dealing with ADHD experienced COVID-19 and to identify potential responses and practical safeguards for future times of crisis. Thus, it was a deliberate aim of the paper to offer practical insights.

**Comment 8:** If the Systems Theory does not work well for the whole storytelling, is there any other theory frameworks in other perspectives that can help? For example, theories in family counseling? I am not familiar with them either, just help brainstorm together.

**Response**: We have incorporated Attachment theory into the paper (page 8 line 178 of the introduction and page 32 line 703 of the discussion). This theory also offers a valuable framework to contextualize the study.

**Comment 9**: Thank you very much for your hard work to make us understand ADHD families in this hard time.

**Response**: Thank you for your thoughtful feedback and suggestions. The recommended changes have significantly strengthened the paper.
