# Supplementary material for: The impact of the COVID-19 pandemic on the mental health of families dealing with attention-deficit hyperactivity disorder
Source: PLoS One. 2023 Mar 16;18(3):e0283227. doi: 10.1371/journal.pone.0283227 (PMC10019744; doi:10.1371/journal.pone.0283227)
Supplement: Attachment — Submitted filename: Response to Reviewer.docx [file pone.0283227.s004.docx]

**Reviewer #3**

Comment 1: Since it is a qualitative research, the findings should be more in-depth.

Response: We disagree that our findings are not in-depth. We have unpacked each theme and subtheme as it relates to previous research and have situated our results within the context of two key theoretical frameworks. We have also included a detailed section that offers practical suggestions for future clinical practice and research.

Comment 2: Would there be any correlation between demographic factors such as family background and educational background with the ADHD child in handling the online teaching at home?

Response: Although this is an interesting question, the suggestion concerns a very specific topic and is beyond the scope of the current research study. To avoid the paper becoming too broad and disorganized we do not think it is appropriate to comment on such niche topics.

Comment 3: In addition, those incidents also happened in normal family such as anxiety and uncertainty during the pandemic.

Response: We have already addressed throughout the manuscript how “normal” families also experienced many adverse situations and we have emphasized in many places how it is imperative to address how the magnitude of the effects of the pandemic on mental health outcomes is amplified in families dealing with ADHD.

Comment 4: From the findings, will the three different group diagnosis needed different level of assistance as no one solution could fit all.

Response: Similar to a comment above, this is an interesting and important point, but it is beyond the scope of the current study. We did not investigate the differences in ADHD diagnoses and whether they would require a different approach.

Comment 5: Furthermore, COVID-19 has come to an end very soon, will it still relevant for addressing the issue.

Response: We have already addressed this concern and indicated that families dealing with ADHD often find themselves in high-stress environments, often mimicking similar features of COVID-19 (isolation, reduced access to resources, lack of support, etc.) Thus, we emphasize that the findings from the current study are applicable beyond the parameters of a pandemic.
